# Evaluation of the thrombus of abdominal aortic aneurysms using contrast enhanced ultrasound - preliminary results

**DOI:** 10.1038/srep34152

**Published:** 2016-09-28

**Authors:** Adam Łukasiewicz, Adam Garkowski, Katarzyna Rutka, Jacek Janica, Urszula Łebkowska

**Affiliations:** 1 Department of Radiology, Medical University of Bialystok, Poland

## Abstract

It is hypothesized that the degree of vascularization of the thrombus may have a significant impact on the rupture of aortic aneurysms. The presence of neovascularization of the vessel wall and mural thrombus has been confirmed only in histopathological studies. However, no non-invasive imaging technique of qualitative assessment of thrombus and neovascularization has been implemented so far. Contrast-enhanced ultrasound (CEUS) has been proposed as a feasible and minimally invasive technique for *in vivo* visualization of neovascularization in the evaluation of tumors and atherosclerotic plaques. The aim of this study was the evaluation of mural thrombus and AAAs wall with CEUS. CEUS was performed in a group of seventeen patients with AAAs. The mural thrombus enhancement was recognized in 12 cases, yet no significant correlation between the degree of contrast enhancement and AAAs diameter, thrombus width, and thrombus echogenicity was found. We observed a rise in AAAs thrombus heterogeneity with the increase in the aneurysm diameter (r = 0.62, p = 0.017). In conclusion CEUS can visualize small channels within AAAs thrombus, which could be a result of an ongoing angiogenesis. There is a need for further research to find out whether the degree of vascularization of the thrombus may have a significant impact on the rupture of aneurysms.

Current attempts in the diagnostic path of abdominal aortic aneurysms (AAAs) are aimed at a better prediction of AAAs progression to help prevent their rupture. The main etiological factor of AAAs is well known decreased strength of the vessel wall with age due to elastic fiber degradation and the transformation of their spatial structure, which results in the vessel dilatation. Damage to vessel wall activates coagulation and thrombosis. A growing thrombus impairs the vessel wall nutrition, causing decreased oxygenation and maldistribution of nutrients from blood to the vessel wall. Recent studies indicate that the initiation and progression of aortic aneurysms may be associated with the presence of mural thrombus, which is the activation site of numerous mediators of inflammation as well as the site of the process of increased angiogenesis^1^,^2^,^3^,^4^,^5^. Impediment to oxygen diffusion to the artery wall caused by a thrombus in the aneurysm sack stimulates the formation of new blood vessels within the thrombus^6^,^7^. It is recognized that the proliferation of a network of small blood vessels in the aneurysm wall and in the thrombus by feeding the inflammatory mediators may be one of the major factors leading to the weakening of the aneurysm wall. Consequently, it results in the progression of the aneurysm size and the increased risk of aneurysm rupture^8^,^9^,^10^.

Modern imaging methods, despite their continual improvement, enable only morphological and qualitative thrombus assessment. The quantitative evaluation of neovascularization in the AAA wall is possible only in the histopathological examination of the preserved tissues sample after AAA surgery^9^.

Although neovascularization of the aneurysm wall and the thrombus has been previously reported, the literature almost exclusively focuses on histopatological assessment of the samples collected during surgery^8^,^10^. As far as we know, there are no reports taking into account standard non-invasive imaging techniques which assess the formation of new blood vessels within wall and mural thrombus of AAAs. Therefore, the focus of this study was to estimate the possibility to visualize newly formed vessels within the mural thrombus as a result of the process of neovascularization using contrast-enhanced ultrasound (CEUS) examination. CEUS with well tolerated contrast agent SonoVue^®^ is a safe and well-established technique for imaging of both solid organs and blood vessels. Unlike the contrast agents used in computed tomography and magnetic resonance imaging, SonoVue^®^ does not diffuse to extravascular space, which allows for the evaluation of even very small blood vessels^11^,^12^. Currently, the use of CEUS focuses mainly on the evaluation of focal lesions, however attempts are now being made to assess the degree of neovascularization within early atherosclerotic lesions and also in the in malignant focal lesions^13^,^14^,^15^,^16^. Taking the above into account and histological evidence of microvessels demonstrated in previous studies within aneurysmal thrombus specimens, CEUS appears to be a promising method for evaluating the degree of neovascularization within AAA thrombus in assessing the risk of AAA rupture.

## Material and Methods

Standard ultrasound and CEUS were performed in 17 patients diagnosed with AAA (15 males, 2 females). The mean age of the patients was 74.1 ± 6.97 years (range, 57–84). The following inclusion criteria were applied: (1) patients with infrarenal AAAs; (2) AAA diameter greater than or equal to 40 mm; (3) the presence of mural thrombus. Exclusion criteria were as follows: (1) contraindications for the administration of contrast agent (SonoVue^®^); (2) previous infrarenal AAA surgery or previous endovascular AAA repair.

Ultrasound examination was performed after a bolus injection of a contrast agent SonoVue^®^ (Bracco, Italy). The contrast agent was administered into an antecubital vein at a dose of 2.4 mL followed by a 10 mL flush of physiological saline. All CEUS examinations were performed by one experienced radiologist using Siemens SONOLINE Elegra with multi-frequency convex probe (2.5–4 MHz) equipped with Ensemble Contrast Imaging (ECI) and Phase Inversion Technology (Siemens Medical Systems, Inc, Ultrasound Group). All patients underwent first gray-scale sonography to measure the size (transverse and longitudinal axis) of the aneurysm. The continuous contrast-enhanced sonographic examination started immediately after contrast injection lasting up to 6 minutes. Full CEUS examination was recorded digitally on hard drive (still images and cine movie). Next, the obtained material was evaluated for qualitative absence or presence of contrast enhancement as well as its severity. The semiquantitative analysis of AAA thrombus enhancement was performed in scale 0–3, wherein 0 meant no visible enhancement, 1 - few vessels, 2 - several vessels, 3 - multiple vessels. Thrombus enhancement was recognized when a tight band areas of increased echogenicity appeared over time after the administration of contrast agent bolus. The following time delay data were obtained: (1) time to lumen enhancement; (2) time between contrast administration and the thrombus enhancement separately in the inner and outer margin of thrombus. During the ultrasound, the following measurements were obtained: diameter of aneurysmal sac and thrombus width. Continuous variables were presented as mean ± SD. The results were analyzed with the statistical Mann-Whitney test. Correlations were determined by Pearson correlation coefficient test. A value of *P* < 0.05 was considered significant.

The study was performed in accordance with the standards of the Declaration of Helsinki. The study protocol was reviewed and approved by the institutional Ethics Committee of the Medical University of Bialystok, Poland. All participants were informed of the study procedures and signed a written informed consent. This research with all experimental protocol was carried out in accordance with the approved guidelines and the guidelines verified and approved by the institutional Ethics Committee of the Medical University of Bialystok. This study was funded by the Medical University of Białystok, Poland - a-not-for-profit organization.

## Results

**Figure 1:**
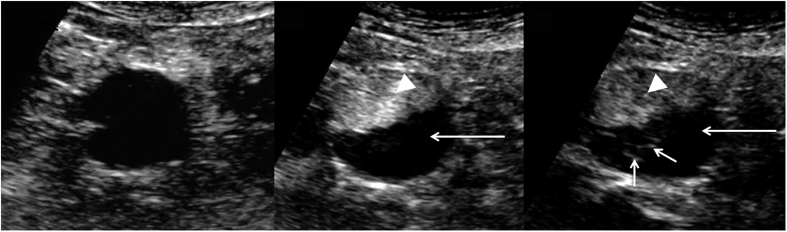
Transverse ultrasound examination of abdominal aortic aneurysm after the administration of contrast agent. (**a**) Ultrasound image at low mechanical index (MI) just before the contrast agent arrival; (**b**) images obtained 15 seconds after administration of the contrast agent, (**b**) images obtained 100 seconds after administration of the contrast agent. The hyperechoic channels within the hypoechoic thrombus are clearly visible (short arrows) (arrowhead - aortic lumen, long arrow-h hypoechoic thrombus).

**Figure 2:**
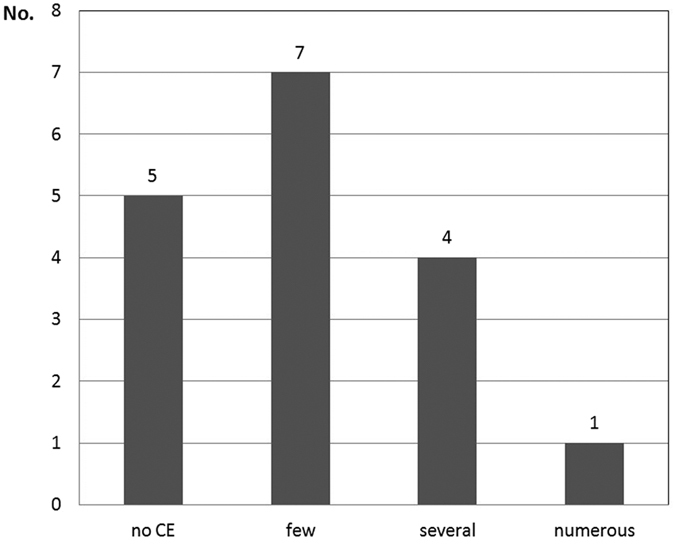
The presence and intensity of thrombus contrast enhancement.

Mean diameter of AAAs was 53.9 ± 14.93 mm (range, 41–98 mm), while mean width of AAAs thrombus was 22.8 ± 5.3 mm (range, 16–36 mm). Table 1 presents the results of the analyzed parameters in the study group. In 12 of 17 patients (70.6%) we found hairy areas of increased echogenicity within mural thrombus after contrast administration (Fig. 1 and Supplementary Video S1). The following outcomes were observed in a semi-qualitative assessment of 12 positive contrast enhancement thrombi: only few visible channels in 7 cases, several channels in 4 cases while numerous channels only in 1 case (Fig. 2). The parameters evaluated in the ultrasonographic examination and in CEUS are shown in Table 2. The enhancement in the lumen of AAAs became apparent 10–25 seconds after contrast injection (mean 16.1 s). The mean time point of lumen-side thrombus enhancement was 40.5 s while the mean timepoint of thrombus enhancement coming from wall-side was about 71 s.

**Figure 3:**
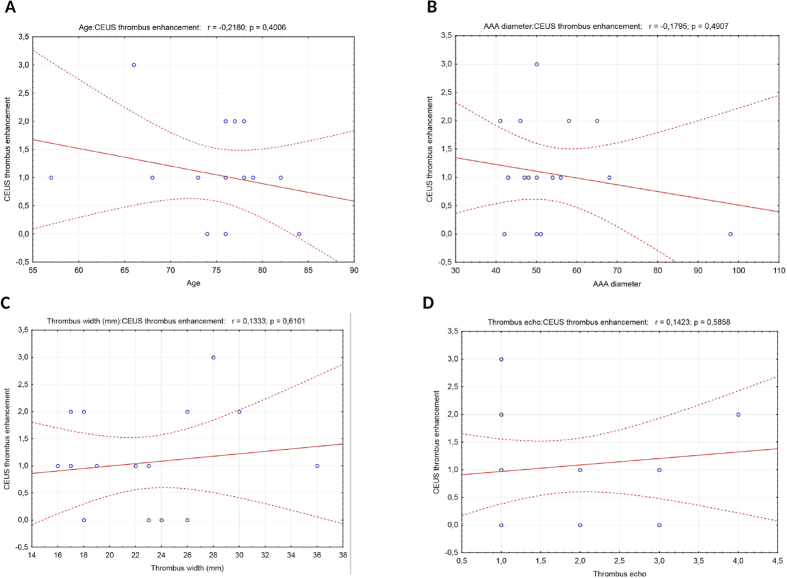
(**A**–**D**) No statistically significant correlation between CEUS thrombus enhancement with age (**A**), AAA diameter (**B**), thrombus width (**C**), and thrombus echogenicity (**D**).

We observed a significantly positive correlation between AAA diameter and thrombus echogenicity – the thrombus heterogeneity rose with the increase in aneurysm diameter (r = 0.62, p = 0.017). There was no significant correlation between the degree of contrast enhancement, age, AAA diameter, thrombus width, and thrombus echogenicity (Fig. 3A–D).

## Discussion

To the best of our knowledge, the present study is one of the first attempts to visualize neovascularization within mural thrombus of AAAs in CEUS. Currently available imaging methods allow for excellent assessment AAA geometry, however, they are of little help in assessing the risk of rupture^17^,^18^. Thrombus is considered as an avascular or very slightly vascularized tissue, and thus the use of contrast agent does not allow for quantitative evaluation of parenchymal perfusion. It is well-known that the ultrasound examinations depend on the examiner and have limited accuracy in obese individuals. These circumstances can interfere examinations and lead to misinterpretations. The morphology of AAA thrombus may be influenced by a number of factors including AAA diameter, width of mural thrombus, and its age, leading to degenerative changes within the thrombus.

Imaging methods with the use of dynamic contrast administration and continuous scanning (e.g., angiography, CT, MRI or CEUS) enable the assessment of the morphology of thrombus and AAA wall. In addition, monitoring thrombus during the administration of contrast agent may provide information about the structural integrity of the thrombus and AAA wall^19^,^20^.

Histopathological evaluation of the affected wall and intraluminal thrombus proves intense angiogenesis and inflammatory process within them^2^,^8^,^21^. Local hypoxia induced by thrombus in aneurysmal sac stimulates many growth factors and cytokines, resulting in the formation of new vessels^5^,^22^,^23^. Kazi *et al*. suggested that inflammatory cells in AAA thrombus may lead to vessel wall impairment and the increased the risk of aneurysm rupture^22^. Our previous results showed inflammatory cells infiltration accompanied by intensive angiogenesis within the AAA thrombus^24^. The proliferation of small vessels within the aneurysm wall and in the intraluminal thrombus may decrease the integrity and stability of AAA wall and be one of the factors responsible for both progression and increased risk of AAA rupture^8^. Inflammation and neovascularization within the thrombus may contribute to enhancement visualization in CEUS examinations^25^. We believe that the demonstration of small channels of contrast enhancement within the thrombus that we found in 11 patients is the result of AAA thrombus vascularization process. In recent studies, CEUS has been reported to assess intraplaque neovascularization and vasa vasorum proliferation^26^,^27^.

It is still uncertain whether the detection of vascular tubules by CEUS corresponds to microvessels within thrombus or plaque. Vavuranakis *et al*. confirmed the presence of microvessels within carotid plaques by immunochemistry (CD34 antibody) and by CEUS examination. Microvessel density in immunochemistry was greater in unstable vs. stable plaques (36.6 ± 17.4 vs. 13.0 ± 7.2 respectively, p = 0.002). Nevertheless, there was no significant correlation between plaque brightness enhancement on CEUS and microvessel density in immunochemistry^27^.

Clevert *et al*. claim that neovascularization and inflammatory changes in the plaque lead to plaque rupture^28^. However, Giannoni *et al*. reported a correlation between contrast enhancement in plaques from symptomatic patients and increased number of microvessels in the histological examination^14^.

Taking into account the results of the studies on the mechanism of plaque vascularization, changes in AAA thrombus may be similar. The traditional morphological description of aneurysmal thrombus (width, echogenicity) is insufficient to predict the risk of rupture. CEUS examination provides a dynamic real-time assessment of perfusion of different kind of tissues and may be useful to detect the vascularization of AAA thrombus. Ultrasound contrast agents are compounds of blood pool which are located in blood vessel and do not diffuse through the walls. This feature allows for visualization of the direct parenchymal perfusion^25^. After the administration of contrast agent in ultrasound, the presence of hyperechogenic areas may suggest the enhancement of the AAA thrombus and the presence of microtubule-developing vessels. The studies confirm that *in vitro* neoangiogenesis, contrast enhancement and stability of the plaque are strongly connected^25^. However, the identification of enhancement during CEUS in AAA thrombus may not always reflect the presence of vascular components, yet the presence of contrast leak through breaks of the thrombus is also possible. The next step should be finding both histological evidence of microvessels within aneurysmal thrombus specimens and the correlation with the findings in CEUS.

There are certain limitations to this study. Firstly, it involved a small number of patients at one institution and thus it is necessary to include more patients in future research. However, a decreasing number of open surgeries to repair AAAs and an increasing number of endovascular AAAs repair make it difficult to gather a sufficient group of patients, and subsequently to evaluate the characteristics of the AAAs with CEUS and compare these findings with post-operative samples. Second, the recognition of the patters of contrast enhancement was based on a subjective index. Furthermore, it is hard to explain different degree of contrast enhancement and lack of enhancement in some cases. Therefore, further research is necessary to answer whether the degree of vascularization of the thrombus may have a significant impact on the aneurysm rupture. Nevertheless, CEUS appears to be one of the promising tools for the assessment of thrombus stability and prediction of aneurysm rupture.

In conclusion, in some cases CEUS may visualize neovascularization in AAA mural thrombus, but there is a need for further evaluation as the correlation between vascularization and rupture risk is still unknown.

## Additional Information

**How to cite this article**: Łukasiewicz, A. *et al*. Evaluation of the thrombus of abdominal aortic aneurysms using contrast enhanced ultrasound - preliminary results. *Sci. Rep.* **6**, 34152; doi: 10.1038/srep34152 (2016).

## Supplementary Material

Supplementary Video S1

Supplementary Information

## Figures and Tables

**Table 1:** Complete list of patients and analyzed parameters.

Patient No.	AAA diameter (mm)	Thrombus width (mm)	Thrombus echo	CEUS thrombus enhancement
1	50	28	1	3
2	42	23	2	0
3	47	17	2	1
4	54	22	2	1
5	58	26	4	2
6	98	23	3	0
7	41	18	1	2
8	46	17	1	2
9	65	30	4	2
10	68	36	3	1
11	50	22	1	1
12	43	19	1	1
13	48	23	1	1
14	50	26	1	0
15	51	24	1	0
16	56	16	1	1
17	50	18	1	0

Thrombus echo: 1- homogenic, 2 - heterogenic, 3 - heterogenic with aechogenic areas, 4 - rupture with flow. CEUS thrombus enhancement: 0 - no visible enhancement, 1 - few vessels, 2 - several vessels, 3 - multiple vessels.

**Table 2:** Parameters evaluated in the examined group.

	Mean	Standard Deviation (SD)
Age (years)	74.1	6.97
AAA diameter (mm)	53.9	14.93
Thrombus thickness (mm)	22.8	5.3
Timepoint of lumen enhancement (s)	16.1	4.94
Timepoint of lumen-side thrombus enhancement (s)	40.5	12.06
Timepoint of wall-side thrombus enhancement (s)	71.0	28.95
Thickness of contrast enhancement (%) (100%-entire thickness)	51	29
Cases of lumen side CE of thrombus	11 (71.43%)	—
Cases of wall side CE of thrombus	5 (35.71%)	—
