# Supplementary material for: Evaluation of the thrombus of abdominal aortic aneurysms using contrast enhanced ultrasound - preliminary results
Source: Sci Rep. 2016 Sep 28;6:34152. doi: 10.1038/srep34152 (PMC5039714; doi:10.1038/srep34152)
Supplement: Supplementary Information [file srep34152-s2.doc]

**Evaluation of the thrombus of abdominal aortic aneurysms using contrast enhanced ultrasound - preliminary results**

Adam Łukasiewicz*, Adam Garkowski, Katarzyna Rutka, Jacek Janica and Urszula Łebkowska

Department of Radiology, Medical University of Bialystok, Poland

Supplementary Video S1. CEUS examination. After 40 and 100 seconds after administration of contrast agent, CEUS shows small hyperechoic channels within the hypoechoic thrombus suggesting neovascularization (arrows).
